# Supplementary material for: Dormancy-specific imprinting underlies maternal inheritance of seed dormancy in Arabidopsis thaliana
Source: eLife. 2016 Dec 28;5:e19573. doi: 10.7554/eLife.19573 (PMC5243116; doi:10.7554/eLife.19573)
Supplement: Supplementary file 1. — DOI: http://dx.doi.org/10.7554/eLife.19573.035 [file elife-19573-supp1.docx]

**Supplementary file1.** List of germination-related MEGs

Germination-related MEGs identified in dormant seed coats.

| **No**. | **Gene ID** | **Gene** | **Cluster** | **Role during germination** | **Mutant phenotype** | **Ref** | **Evidence** |
| --- | --- | --- | --- | --- | --- | --- | --- |
| 1 | AT2G03760 | AtSOT1, AtSOT12, ATST1, RAR047, SOT12, ST, ST1, sulphotransferase 12 | 1 | Negative regulator of ABA signaling in seeds. | Germination hypersensitive to NaCl and ABA | Baek et al., 2010 | **Direct** |
| 2 | AT2G18980 | Peroxidase superfamily protein | 1 | Negative regulator of dormancy release. Reactive Oxygen Species (ROS) play key roles in dormancy release and seed germination. Peroxidase activity accompanies endosperm rupture in tomato. H_2_O_2_ inhibits ABA-dependent repression of endosperm rupture. | Seeds less dormant than WT | Linkies et al., 2010  El-Maarouf-Bouteau et al., 2008  Morohashi, 2002  Muller et al., 2009 |  |
| 3 | At4g01360 | BYPASS1-related protein, BPS3, BYPASS3 | 1 | Positive regulator of germination. *Arabidopsis* mutants with defects in the *BYPASS1* (*BPS1*) gene overproduce an active mobile compound that moves from the root to the shoot and inhibits growth. | Triple *bps1/bps2/bps3* mutant germinates less well than WT | Lee et al., 2012 |  |
| 4 | AT4G15230 | ATPDR2, PDR2, pleiotropic drug resistance 2 | 1 | Negative regulator of germination. ABA transporter that controls Arabidopsis seed germination. | Seeds less dormant | Kang et al., 2015 |  |
| 5 | AT4G36880 | CP1, cysteine proteinase1 | 1 | Positive regulator of germination. Germination-specific cysteine protease expressed during the early phase of germination, involved in CRCs degradation in Arabidopsis endosperm. | Delayed CRCs mobilization | Tsuji et al., 2012  This study |  |
| 6 | AT5G39340 | AHP3, ATHP2, histidine-containing phosphotransmitter 3 | 2 | Positive regulator of germination. Involved in cytokinin-antagonized ABA effects on cotyledon greening. | Double mutant hypersensitive to ABA during germination | Guan et al., 2014  Nishiyama et al., 2013 |  |
| 7 | AT5G64620 | ATC/VIF2, C/VIF2, cell wall / vacuolar inhibitor of fructosidase 2 | 2 | Negative regulator of germination. Arabidopsis cell wall invertase inhibitor AtCIF1 plays a role in seed germination and early seedling growth. | Insensitive to ABA during germination | Su et al., 2016 |  |

| **No**. | **Gene ID** | **Gene** | **Cluster** | **Potential role during germination** | **Mutant phenotype** | **Ref** | **Evidence** |
| --- | --- | --- | --- | --- | --- | --- | --- |

| 8 | AT1G08920 | ESL1, ERD (early response to dehydration) six-like 1 | 1 | *ESL1* is induced by osmotic stress and ABA. | ND | Kiyosue et al., 1994  Yamada et al., 2010 | **Indirect** |
| --- | --- | --- | --- | --- | --- | --- | --- |
| 9 | AT1G62200 | NRT1/ PTR FAMILY, putative peptide/nitrate transporter | 1 | Nitrate releases seed dormancy in Arabidopsis by lowering ABA levels in imbibed seeds. | ND | Bethke et al., 2006  Matakiadis et al., 2009  Osuna et al., 2015  Albertos et al., 2015 |  |
| 10 | AT2G28550 | RAP2.7, TOE1, related to AP2.7 | 1 | AP2 TFs participate in ABA and osmotic stress signaling. AP2 domains are found in EREBP (ethylene responsive element binding protein). Ethylene interferes with ABA signaling. TOE1 binds to the *FT* promoter regulating *FT* mRNA. | ND | Zhang et al., 2015 |  |
| 11 | AT3G09190 | Concanavalin A-like lectin family protein | 2 | Arabidopsis A4 subfamily of lectin receptor kinases negatively regulates ABA responses in seed germination. | ND | Xin et al., 2009 |  |
| 12 | AT2G46650 | ATCB5-C, B5 #1, CB5-C, cytochrome B5 isoform C | 1 | Sucrose transporter SUT4 interacts with Cyb5 to mediate sucrose and glucose signaling in the sucrose/glucose-induced inhibition of seed germination. | ND | Li et al., 2012 |  |
| 13 | AT4G04955 | ALN, ATALN, allantoinase | 1 | Allantoinase regulates abiotic stress tolerance through activation of abscisic acid metabolism | ND | Watanabe et al., 2014 |  |
| 14 | AT4G30280 | ATXTH18, XTH18, xyloglucan endotransglucosylase/hydrolase 18 | 1 | GA induces XTHs in the micropylar endosperm during tomato seed germination to facilitate endosperm weakening. Induced in DELLA-dependent manner in Arabidopsis during germination. | ND | Chen et al., 2002  Cao et al., 2006 |  |
| 15 | At5g64120 | Peroxidase superfamily protein, AtPRX71 | 1 | AtPRX71 contributes to strengthen cell walls, therefore restricting cell expansion, during normal growth and in response to cell wall damage | ND | Raggi et al., 2015 |  |
| 16 | AT2G16660 | Major facilitator superfamily protein | 1 | Response to karrikins, seed germination stimulants found in smoke from wild fires. Target of PIF1 during germination. | ND | Nelson et al., 2010  Oh et al., 2009 |  |
| 17 | AT1G05000 | Phosphotyrosine protein phosphatases superfamily protein | 2 | Phosphotyrosine phosphatases are involved in ABA-dependent responses in Arabidopsis seeds | ND | Alonso-Ramiréz et al., 2011  Ghelis et al., 2008  Liu et al., 2015  Quettier et al., 2006  Reyes et al., 2006 |  |

ND- not determined

1. Alonso-Ramiréz et al., 2011. Functional analysis in Arabidopsis of FsPTP1, a tyrosine phosphatase from beechnuts, reveals its role as a negative regulator of ABA signaling and seed dormancy and suggests its involvement in ethylene signaling modulation. Planta 234:589–597
2. Ghelis et al., 2008. Protein tyrosine kinases and protein tyrosine phosphatases are involved in abscisic acid-dependent processes in Arabidopsis seeds and suspension cells. Plant Physiol. 148(3): 1668-80.
3. Liu et al., 2015, AtDsPTP1 acts as a negative regulator in osmotic stress signalling during Arabidopsis seed germination and seedling establishment. J Exp Bot. 66(5): 1339-53.
4. Quettier et al., 2006. The phs1-3 mutation in a putative dual-specificity protein tyrosine phosphatase gene provokes hypersensitive responses to abscisic acid in Arabidopsis thaliana. Plant J. 47(5): 711-9.
5. Reyes et al., 2006. Evidence of a role for tyrosine dephosphorylation in the control of postgermination arrest of development by abscisic acid in Arabidopsis thaliana L. Planta 223(2): 381-5.
6. Kiyosue et al., 1994. Cloning of cDNAs for genes that are early-responsive to dehydration stress (ERDs) in Arabidopsis thaliana L.: identification of three ERDs as HSP cognate genes. Plant Mol Biol. 25(5): 791-8.
7. Yamada et al., 2010. Functional analysis of an Arabidopsis thaliana abiotic stress-inducible facilitated diffusion transporter for monosaccharides. J Biol Chem. 285(2): 1138-46.
8. Bethke et al., 2006. Nitric oxide reduces seed dormancy in Arabidopsis. J Exp Bot. 57(3): 517-26.
9. Matakiadis et al., 2009. The Arabidopsis abscisic acid catabolic gene CYP707A2 plays a key role in nitrate control of seed dormancy. Plant Physiol. 149(2):949-60.
10. Osuna D et al., 2015. Control of Seed Germination and Plant Development by Carbon and Nitrogen Availability. Front Plant Sci. 18;6: 1023.
11. Albertos et al., 2015. S-nitrosylation triggers ABI5 degradation to promote seed germination and seedling growth. Nat Commun. 6:8669.
12. Baek et al., 2010. A stress-inducible sulphotransferase sulphonates salicylic acid and confers pathogen resistance in *Arabidopsis*. Plant, Cell & Environment, 33: 1383–1392.
13. Nelson et al., 2010. Karrikins enhance light responses during germination and seedling development in *Arabidopsis thaliana* PNAS April 13, 2010 vol. 107 no. 15 7095-7100
14. Oh et al., 2009 Genome-Wide Analysis of Genes Targeted by PHYTOCHROME INTERACTING FACTOR 3-LIKE5 during Seed Germination in *Arabidopsis* The Plant Cell February 2009 vol. 21 no. 2 403-419
15. Linkies et al., 2010. Peroxidases identified in a subtractive cDNA library approach show tissue-specific transcript abundance and enzyme activity during seed germination of *Lepidium sativum*. *Journal of Experimental Botany*.
16. El-Maarouf-Bouteau et al., 2008. Oxidative signaling in seed germination and dormancy. *Plant Signaling & Behavior*. 2008;3(3):175-182.
17. Morohashi 2002. Peroxidase activity develops in the micropylar endosperm of tomato seeds prior to radicle protrusion. J. Exp. Bot. (2002) 53 (374): 1643-1650.
18. Muller et al., 2009. In Vivo Cell Wall Loosening by Hydroxyl Radicals during Cress Seed Germination and Elongation Growth. Plant Physiology August 2009 vol. 150 no. 4 1855-1865
19. Zhang et al., 2015. *Arabidopsis* TOE proteins convey a photoperiodic signal to antagonize CONSTANS and regulate flowering time. Genes & Dev. 2015. 29: 975-987
20. Xin et al., 2009. The Arabidopsis A4 Subfamily of Lectin Receptor Kinases Negatively Regulates Abscisic Acid Response in Seed Germination . Plant Physiology January 2009 vol. 149 no. 1 434-444
21. Li et al., 2012. *Arabidopsis* Sucrose Transporter SUT4 Interacts with Cytochrome *b5*-2 to Regulate Seed Germination in Response to Sucrose and Glucose. Molecular Plant [Volume 5, Issue 5](http://www.sciencedirect.com/science/journal/16742052/5/5), September 2012, Pages 1029–1041
22. Lee et al., 2012. In the absence of BYPASS1-related gene function, the *bps* signal disrupts embryogenesis by an auxin-independent mechanism. Development139: 805-815;
23. Watanabe et al., 2014. The purine metabolite allantoin enhances abiotic stress tolerance through synergistic activation of abscisic acid metabolism. Plant Cell Environ, 37: 1022–1036
24. Kang et al., 2015. Abscisic acid transporters cooperate to control seed germination. Nat Commun. 2015 Sep 3;6:8113.
25. Chen et al., 2002. A gibberellin-regulated xyloglu- can endotransglycosylase gene is expressed in the endosperm cap during tomato seed germination. J Exp Bot 53:215–223.
26. Cao et al., 2006. Gibberellin Mobilizes Distinct DELLA-Dependent Transcriptomes to Regulate Seed Germination and Floral Development in Arabidopsis. Plant Physiology, Vol. 142, pp. 509–525.
27. Tsuji et al., 2012. Enzymatic characterization of germination-specific cysteine protease-1 expressed transiently in cotyledons during the early phase of germination. J Biochem (2013) 153 (1): 73-83.
28. Guo et al., 2013. Smoke-derived karrikin perception by the α/β-hydrolase KAI2 from *Arabidopsis.* PNAS vol. 110 no. 20 8284-8289
29. Waters et al., 2015. A *Selaginella moellendorffii* Ortholog of KARRIKIN INSENSITIVE2 Functions in Arabidopsis Development but Cannot Mediate Responses to Karrikins or Strigolactones. The Plant Cell vol. 27 no. 7 1925-1944
30. Raggi et al., 2015. The Arabidopsis Class III Peroxidase AtPRX71 Negatively Regulates Growth under Physiological Conditions and in Response to Cell Wall Damage. Plant Physiology December 2015 vol. 169 no. 4 2513-2525
31. Su et al., 2016. Reassessment of an *Arabidopsis* cell wall invertase inhibitor AtCIF1 reveals its role in seed germination and early seedling growth. Plant Mol Biol (2016) 90:137–155

Germination-related MEGs identified in non-dormant seed coats.

| **No**. | **Gene ID** | **Gene** | **Role during germination** | **Mutant phenotype** | **Ref** | **Evidence** |
| --- | --- | --- | --- | --- | --- | --- |
| 1 | At3g44730 | ATKP1, KP1, kinesin-like protein 1 | Involved in respiratory regulation during seed germination at low temperature | When grown at 4°C, KP1 dominant-negative mutants exhibit a higher seed germination frequency. *kp1* mutants had increased oxygen consumption during germination | Yang et al., 2011 | **Direct** |
| 2 | At5g63080 | HR demethylase JMJ20, JMJ20, JUMONJI DOMAIN_CONTAINING PROTEIN 20 | Positive regulator of seed germination in the PHYB-PIL5-SOM pathway | Double mutant jmj20/jmj22 germinates less well than WT in phyB-dependent seed germination | Cho et al., 2012 |  |
| **No**. | **Gene ID** | **Gene** | **Potential role during germination** | **Mutant phenotype** | **Ref** | **Evidence** |
| 3 | At3g04080 | APY1, ATAPY1, apyrase 1 | Play a role in polar auxin transport. ABA represses growth of Arabidopsis embryonic axis by enhancing auxin signaling | ND | Liu et al., 2012  Belin et al., 2009 | **Indirect** |

ND- not determined

1. Liu et al., 2012. Role for Apyrases in Polar Auxin Transport in Arabidopsis. Plant Physiology vol. 160 no. 4 1985-1995
2. Belin et al., 2009. Abscisic Acid Represses Growth of the *Arabidopsis* Embryonic Axis after Germination by Enhancing Auxin Signaling. The Plant Cell vol. 21 no. 8 2253-2268
3. Yang et al., 2011. *Arabidopsis* Kinesin KP1 Specifically Interacts with VDAC3, a Mitochondrial Protein, and Regulates Respiration during Seed Germination at Low Temperature. The Plant Cell vol. 23 no. 3 1093-1106
4. Cho et al., 2012. Control of Seed Germination by Light-Induced Histone Arginine Demethylation Activity. Developmental Cell [Volume 22, Issue 4](http://www.sciencedirect.com/science/journal/15345807/22/4), 17 April 2012, Pages 736–748
